# Association of anemia with sensorineural hearing loss: a systematic review and meta-analysis

**DOI:** 10.1186/s13104-019-4323-z

**Published:** 2019-05-23

**Authors:** Shimel Hussien Mohammed, Sakineh Shab-Bidar, Samer Abuzerr, Tesfa Dejenie Habtewold, Shahab Alizadeh, Kurosh Djafarian

**Affiliations:** 1 Department of Community Nutrition, School of Nutritional Sciences and Dietetics, Tehran University of Medical Sciences-International Campus, Tehran, Iran; 2 Department of Community Nutrition, School of Nutritional Sciences and Dietetics, Tehran University of Medical Sciences, Tehran, Iran; 3 Department of Environmental Health Engineering, Faculty of Public Health, Tehran University of Medical Sciences-International Campus, Tehran, Iran; 4 Department of Nursing, Debre Berhan University, Debre Berhan, Ethiopia; 5 Department of Clinical Nutrition, School of Nutritional Sciences and Dietetics, Tehran University of Medical Sciences, Tehran, Iran

**Keywords:** Anemia, Hearing loss, Hearing impairment, Sensorineural hearing loss, Review

## Abstract

**Objective:**

Evidence shows that anemic individuals are at a higher risk of hearing loss. However, there is no systematic review and meta-analysis study. Thus, we aimed to meta-analyze the existing evidence on the association of iron deficiency anemia (IDA) with sensorineural hearing loss (SNHL). We searched PubMed, MEDLINE, Embase, Scopus, and Google Scholar from inception through October 30, 2017, for studies done on the association of the IDA with SNHL. Pooled odds ratio (OR) was calculated by random effect meta-analysis method. Heterogeneity was assessed by I^2^ metrics.

**Result:**

Four studies, covering a total of 344,080 adults and children, were included. The odds of SNHL was higher by 55% in individuals with IDA, compared with individuals without IDA (OR = 1.55, 95% CI 1.17–2.06; P = 0.03). The age-specific ORs were 1.36 (95% CI 1.15–1.61; P = 0.27) and 3.67 (95% CI 1.72–7.84) for adults and children, respectively. IDA may be a contributing factor to hearing loss. Further studies are warranted, including whether IDA treatment reduces the risk of hearing loss. Meanwhile, hearing loss screening in anemic individuals, or vice versa, may represent an important consideration.

*PROSPERO registration* CRD42017082108

**Electronic supplementary material:**

The online version of this article (10.1186/s13104-019-4323-z) contains supplementary material, which is available to authorized users.

## Introduction

Anemia remains among the major global public health problems, affecting almost a third of the world population (over 2.2 billion people) [1, 2]. Though it is a multifactorial problem, iron deficiency anemia (IDA) is estimated to account for almost half of the global anemia burden. There is a wide variation in the distribution of IDA, with the highest prevalence being in central Asia (64.7%) and lowest in North America (2.9%) [3–5]. Hearing loss is also a significant public health challenge of a high, but less recognized, magnitude [6]. Although the figures may vary with the hearing assessment methods and thresholds used, hearing loss was estimated to affect 360 [7] to 554 [8] million individuals globally. Hearing loss is an important contributor to childhood disability [9]. Hearing loss is of negative consequences, including poor psychosocial, cognitive and language developments in children [10] and cognitive decline, dementia, and social isolation in the elderly [11–14].

Existing evidence showed IDA linked to hearing loss. A higher prevalence of hearing loss was reported in individuals with IDA [15, 16]. The exact mechanism of IDA leading to hearing loss is mainly unclear. Whether IDA treatment improves hearing function is also unclear. However, it has been widely hypothesized to be related to the sensitivity of the cochlea to vascular and neurologic effects of IDA. Cochlea is supplied only by the labyrinthine artery. The lack of collateral circulation could make it more vulnerable to the ischemic effect of IDA [15–19]. To the best of our knowledge, there is no systematic review and meta-analysis report on the association of anemia and hearing impairment. We, therefore, conducted this systematic review and meta-analysis on the link of IDA to sensorineural hearing loss (SNHL).

## Main text

### Methods

This systematic review and meta-analysis was conducted following the recommendations of Meta-analysis of Observational Studies in Epidemiology guideline [20], and the Preferred Reporting Items for Systematic Reviews and Meta-Analyses (PRISMA) [21]. The work was registered in PROSPERO.

#### Search strategy and data sources

We searched PubMed, MEDLINE, Embase, Scopus, and Google Scholar for studies published in English from inception to June 30, 2018. We used a combination of search terms including ‘anemia’, ‘anaemia’, ‘iron deficiency anaemia’, ‘iron deficiency anemia’, ‘iron-deficiency anaemia’, ‘iron deficiency anemia’, ‘hearing loss’, ‘deafness’, ‘hearing impairment’, and ‘hearing dysfunction’.

#### Eligibility criteria

The inclusion criteria were (1) hearing loss measured and reported, (2) IDA measured and reported, and (3) adjusted OR, incidence rate (IR), or relative risk (RR) reported on the association of anemia and hearing loss. The exclusion criteria were any one of the following conditions: (1) animal studies; (2) celiac disease; (3) anemia of causes specified other than iron deficiency; or (4) qualitative studies, commentaries, editorials, and case reports.

#### Study screening and data extraction

Literature search, title, abstract and full-text screening and reviewing were done by SHM and SA, working independently and in duplicate. The screening and selection processes are illustrated using the PRISMA flow diagram (Additional file 1). From each study, we extracted the following information: (1) study identification (title, first author, year of publication), (2) study characteristics (country, study design, sample size, follow-up period for longitudinal studies), (3) participants’ demographic factors (mean age, proportion of men), (4) IDA assessment method, (5) hearing loss assessment method, (6) measure of anemia and hearing loss association, and (7) variables used for adjustments. The pre-specified measures of association were adjusted OR, RR, or IR of hearing loss in individuals with IDA, compared with those without IDA. When a study reported more than one OR/RR, we took the maximum adjusted one that was adjusted for more variables. Besides, we treated RR as the equivalent of OR. For studies in which the reference of comparison was the risky group (individuals with IDA), we took the inverse of the reported estimate to ensure all estimates use the same reference group (individuals without IDA).

#### Quality assessment

We assessed the methodological quality of included studies using the Newcastle–Ottawa Scale (NOS) [22]. The grading was out of 9 and a score 0 to 3 was considered low quality, 4 to 6 medium quality, and 7–9 high quality. Ratings for each study were compared between the two evaluators, SHM and SA, with discrepancy resolved by consensus.

### Statistical analysis

The OR was used to summarise the estimates, representing the odds of hearing loss in those with IDA, compared with those without IDA. The summary OR was calculated with the DerSimonian and Laird random effect model, which accounts for both within study and between studies variations [23]. Heterogeneity between studies was assessed using the I^2^-statistics, which quantifies the proportion of variance explained by between-study heterogeneity. According to Higgins et al. [24], I^2^ < 25%, 25–49%, 50–75%, and > 75% represents no, low, moderate and high levels of heterogeneity, respectively. Potential sources of heterogeneity were identified using subgroup analyses. We intended to assess publication bias using the funnel plot technique, Begg’s rank test, and Egger’s regression test, as appropriate. However, it was not possible as there were inadequate numbers of studies, which under-power any of these methods. A minimum of 10 studies is recommended to evaluate publication bias, although a meta-analysis of 2 or more studies is possible [25]. Stata 15.0 was used for all analyses.

### Result

We included a total of four studies [15–18] in this work. The literature search, selection and reviewing process is shown in the PRISMA flow diagram (Additional file 1). The general characteristic of the studies is presented in Table 1. The studies were published from 2011 to 2017. Of the four studies included in this work, three were cohort and one was case–control. Except one study done in Taiwan, the remaining three studies were conducted in the USA. The quality of the studies was high, all scoring > 7/9 on the NOS quality assessment tool. The sample size of the studies ranged from 2612 to 305,339 individuals. Altogether, the four studies included 344,080 unique individuals, of whom 49.60% were males (n = 151,461) and 50.40% females (n = 153,878). The mean age of participants was 48.2 years. Of the four studies, three were done on adults and one on children and adolescents.

**Table 1:** Main characteristics of studies included in this meta-analysis

First Author, Publication year	Setting	Population	Study design	Sample size	Quality score	IDA^a^ Measure^a^	Hearing loss Measure
Schieffer 2017 [15]	USA	Children	Cohort	20,113	9	Serum ferritin Hemoglobin	SNHL^c^
Schieffer 2017 [17]	USA	Adults	Cohort	305,339	9	Serum ferritin Hemoglobin	SNHL
Chung 2014 [18]	Taiwan	Adults	CC^b^	16,016	8	Serum ferritin Hemoglobin	SNHL
Nash 2011 [16]	USA	Adults	Cohort	2612	9	Hematocrit	SNHL

^a^Iron deficiency anemia; ^b^CC = case–control; ^c^SNHL = Sensorineural hearing loss

**Fig. 1:**
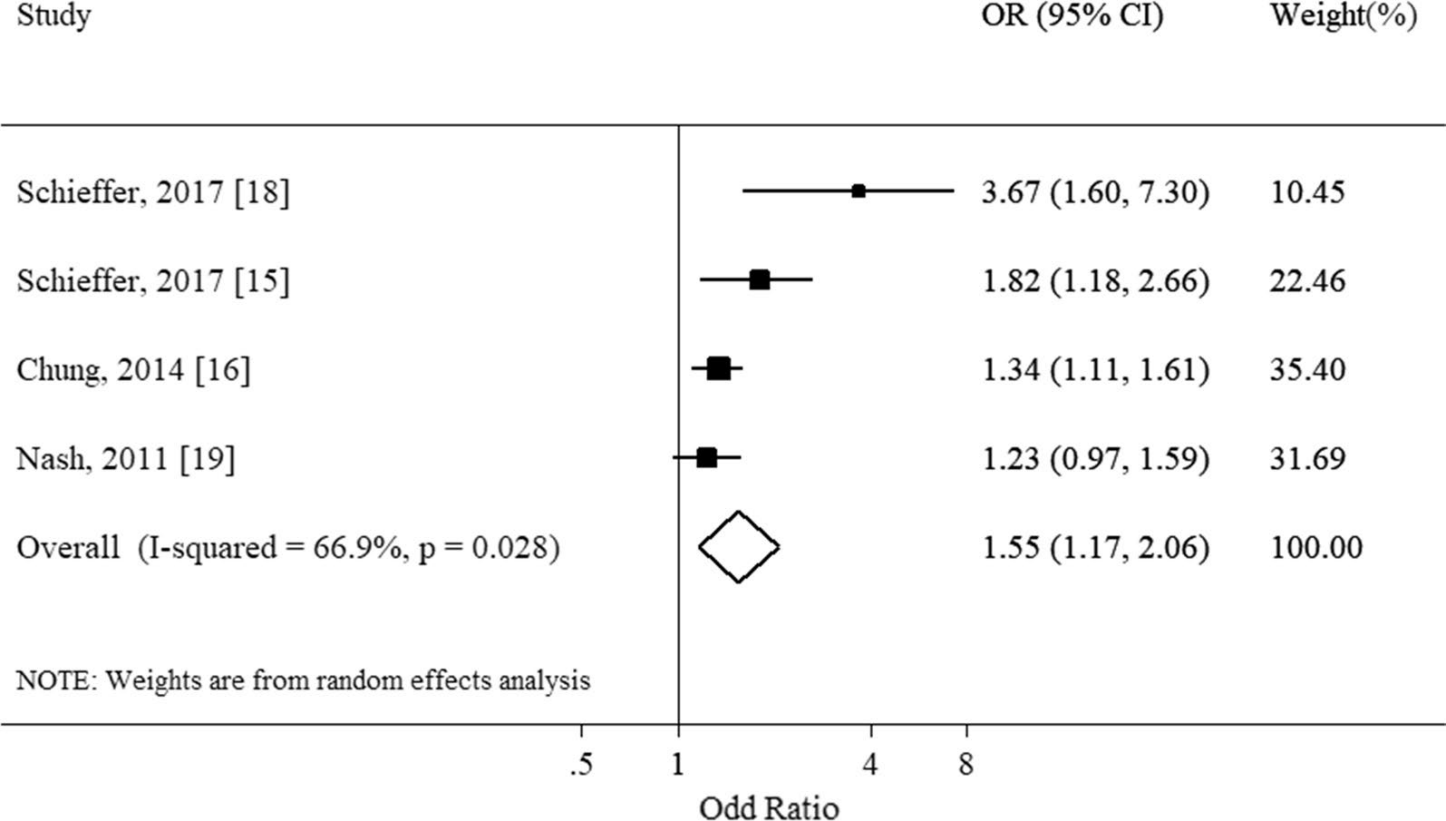
Association of iron deficiency anemia and sensorineural hearing loss

**Fig. 2:**
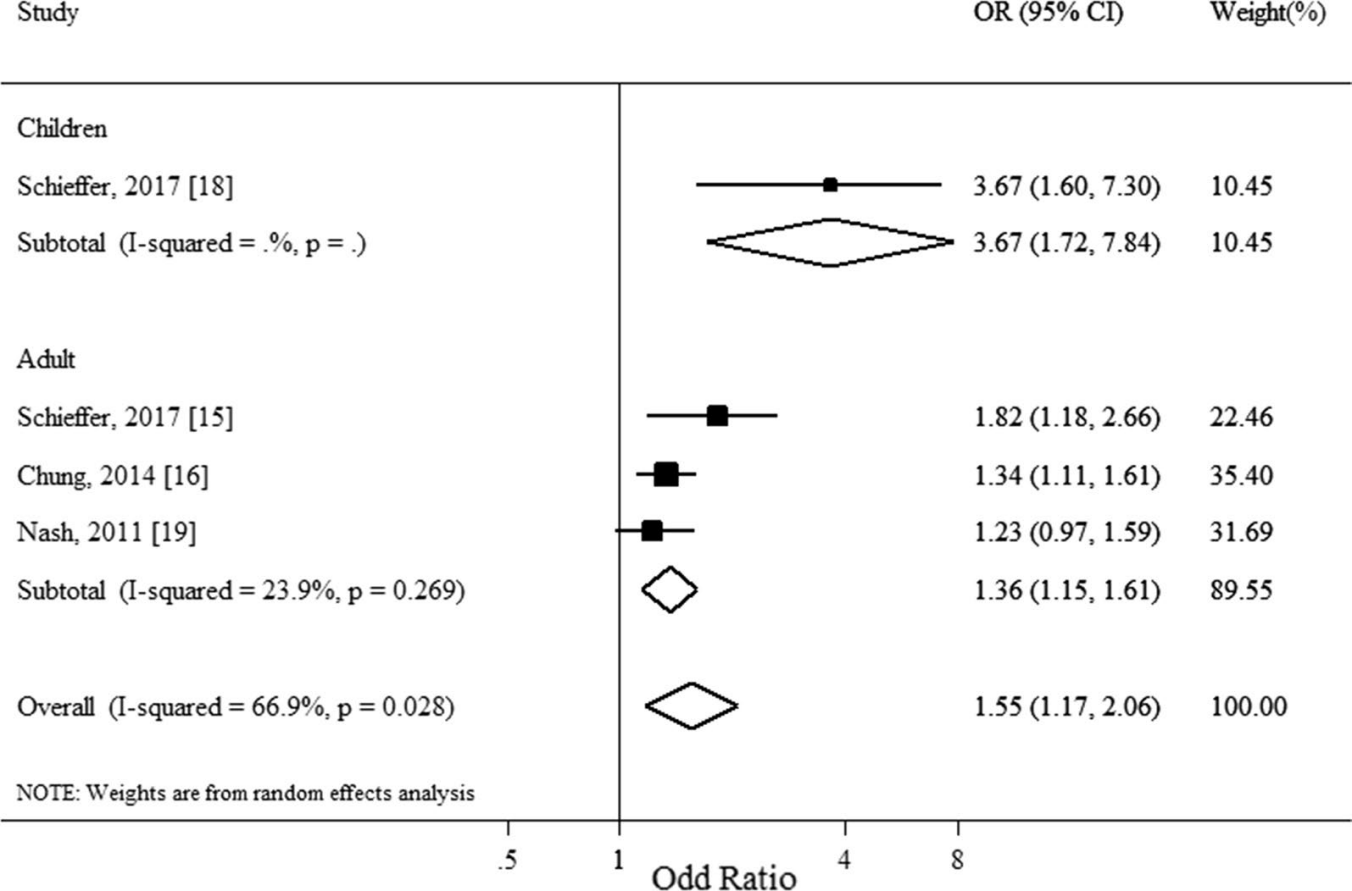
Subgroup analysis of association iron deficiency anemia and sensorineural hearing loss

Figure 1 shows the summary estimate of the association of IDA with hearing loss, calculated combining all the studies with random effect model. The estimates (ORs) refer to the odds of hearing impairment among anemic cases, as compared with non-anemic ones. It showed IDA significantly associated with hearing loss (pooled OR = 1.55, 95%CI 1.17-2.06) with a moderate level of heterogeneity (Q = 9.06, df = 3; I^2^ = 66.9%, P = 0.03 for heterogeneity). Subgroup analysis was conducted by age groups, separating studies on adult and children. Figure 2 shows the results of the subgroup analyses. The child study seemed to explain the heterogeneity between the studies. Exclusion of this study resulted in a lower level of heterogeneity (Adult: Q = 2.63, df = 2; I^2^ = 23.9%, P = 0.27), but the associations remained significant and almost the same (pooled OR = 1.55, 95 CI% = 1.17–2.06 versus 1.36, 95% CI 1.15–1.61; with and without the child study included). Thus, the summary estimate of odds of SNHL associated with IDA was 1.36 (95% CI 1.15–1.61; I^2^ = 23.9, P = 0.27) in adults and 3.67 (95% CI 1.72–7.84) in children and adolescents.

### Discussion

We found a significant association between IDA and SNHL among both adults and children. The odds of SNHL was 55% higher in individuals with IDA, compared with those without IDA. A recent study in the USA showed that the prevalence of hearing impairment was 3.0% in children with IDA, but 1.7% in those without IDA [15]. In general, hearing impairment was estimated to affect 10% of the world population in the year 2015 [2], with significant variations across age groups as well as geographic regions. In 2011, 20.6% American adults aged 48–59 years and 90% of those older than 80 years were reported to have hearing impairment [16], with an estimated overall adult prevalence of 14.1% [17].

The mechanism linking IDA and SNHL is uncertain and non-conclusive, through some plausible mechanisms were proposed. A more frequently mentioned mechanism is the vascular hypothesis that the cochlea is susceptible to ischemia due to lack of collateral circulation. Provided iron is essential for the production of hemoglobin, one could presume IDA will likely compromise tissue oxygen delivery [3, 4, 18] and increase the risk of ischemia [18, 26]. Cochlea receives blood from only the labyrinthine artery and would be more vulnerable to a reduction in blood oxygen due to IDA. Another proposed mechanism is the role of iron in the nervous system. Iron is a cofactor in neurotransmitter metabolism, DNA synthesis, and nerve myelination [27, 28]. Neurological disorders like epilepsy, peripheral neuropathy, myoclonus, posterior column demyelination, headache, cerebellar ataxia, brain atrophy, and dementia might be linked to SNHL. Celiac disease has also been associated with hearing impairment, due to reasons including impairment in nutritional status, diarrhea, abdominal distension and weight loss [29].

Anemia and hearing loss are among the top leading contributors to health impairment globally [2]. Anemia prevention and control is a global priority agenda [5]. Unlike anemia, hearing loss is in the rank of neglected problems, with low public health attention [6, 7]. Studies on IDA and hearing loss are limited in number as well as geographic coverage. The existing estimates are based mainly on studies conducted in western countries, where prevalence IDA is low and the possibility of early diagnosis and treatment is better, compared with the regions of Asia and Africa where both IDA and hearing loss burdens are high [8]. Thus, presuming IDA might be a risk factor for hearing loss, we believe that the number of people with hearing loss may be even higher than the reported figures in the literature. Further studies from developing countries would contribute to further understanding the nature and extent of the association of the two conditions.

While IDA remains a major public health problem globally, its association with hearing loss would be more concerning. The existing evidence is limited on whether anemia prevention and treatment reduces the risk of hearing loss or treatment of anemia in individuals with concurrent anemia and hearing impairment improves hearing function. Although the design of the studies included in our work precluded making a causal inference and definitive conclusion, we suggest further studies on the feasibility and effectiveness of hearing loss screening for individuals with anemia or anemia screening for individuals with hearing loss. Prevention, early diagnosis, and treatment of IDA could have dual benefits, reducing the burden of anemia and probably hearing loss. Previous reports also recommended the importance of screening [15, 17, 18]. Consideration of hearing loss screening for anemic individuals may stand more important in settings with high anemia prevalence. Strategies for integrating hearing loss screening for anemic cases, or vice versa, into existing public health and clinical practices need to be explored by further studies. Anemia prevention and control is a centrality in national and global nutrition programs, with established delivery platforms. Thus, integrating hearing loss with anemia prevention and control delivery platforms may be an efficient option, albeit it should be confirmed in future studies.

### Conclusion

This study showed a significant association between IDA and hearing loss. The exact mechanism by which IDA contributes to hearing loss, and whether IDA treatment reduces the risk of hearing loss is unclear. Further studies are warranted to better understand the link between IDA and hearing loss and draw public health attention. Besides, the potential of integrating hearing loss screening for anemic patients, or vice versa, needs to be further explored.

## Limitations

- We found only four studies, which underpowered any of the existing publication bias evaluation methods.
- Except one study done from Taiwan, the other studies refer only to the US population, limiting the generalizability of the findings to the rest of the world.
- All studies were observational in design, precluding making causal inferences.

## Additional file

**Additional file 1.** PRISMA flow diagram of study selection and screening.

## Data Availability

The study is based on extracting data from published articles and all data are included in the report.

## Abbreviations

CI: confidence interval
IDA: iron deficiency anemia
OR: odds ratio
RR: relative risk
SNHL: sensorineural hearing loss
WHO: World Health Organization
