# Supplementary figures and images for: Association of anemia with sensorineural hearing loss: a systematic review and meta-analysis
Source: BMC Res Notes. 2019 May 23;12:283. doi: 10.1186/s13104-019-4323-z (PMC6533653; doi:10.1186/s13104-019-4323-z)

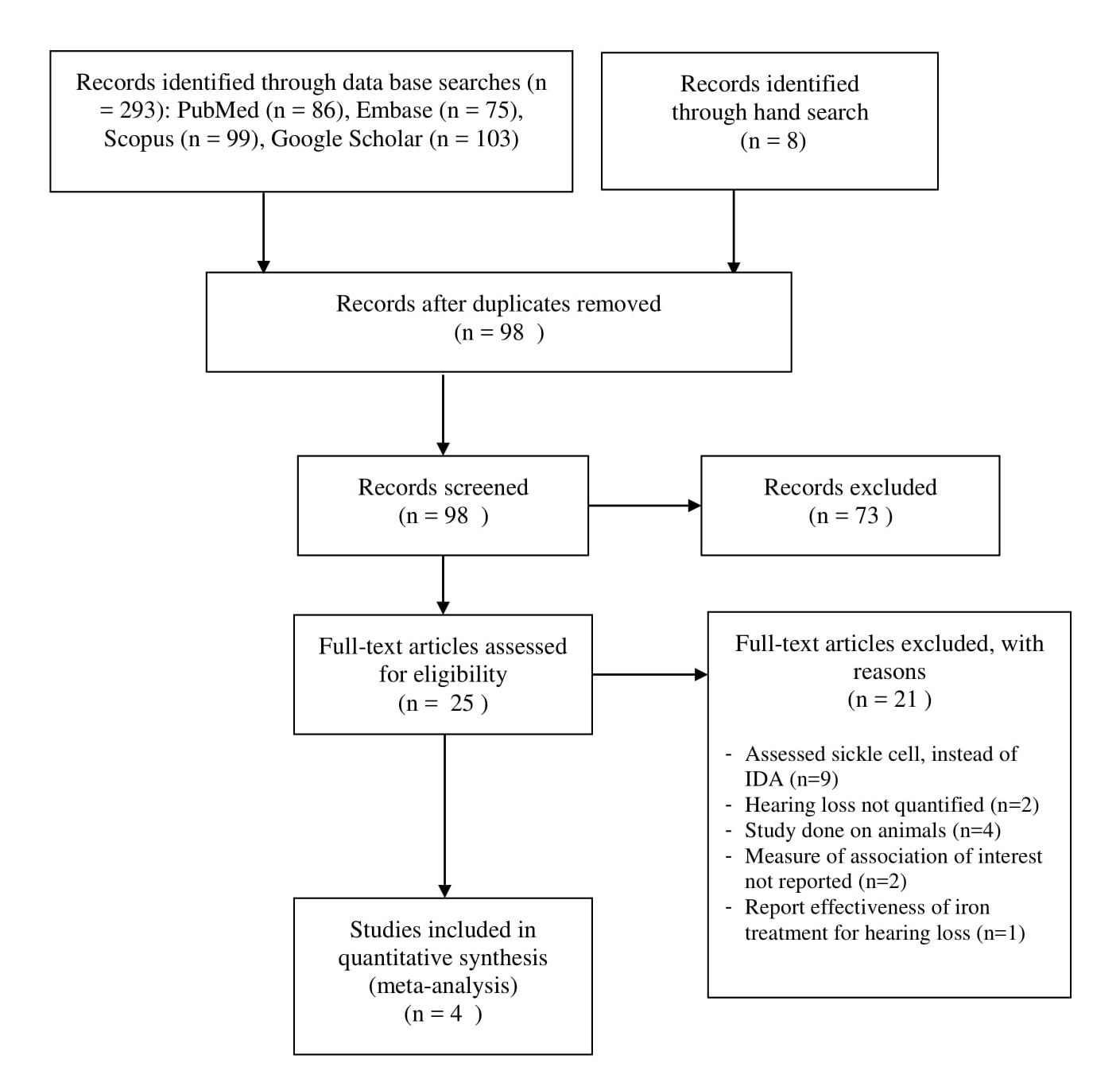

Supplement: Supplementary file 1 — Additional file 1. PRISMA flow diagram of study selection and screening. [file 13104_2019_4323_MOESM1_ESM.tif]
